# Seroprevalence of SARS-CoV-2 in four states of Nigeria in October 2020: A population-based household survey

**DOI:** 10.1371/journal.pgph.0000363

**Published:** 2022-06-17

**Authors:** Rosemary A. Audu, Kristen A. Stafford, Laura Steinhardt, Zaidat A. Musa, Nnaemeka Iriemenam, Elsie Ilori, Natalia Blanco, Andrew Mitchell, Yohhei Hamada, Mirna Moloney, Emem Iwara, Alash’le Abimiku, Fehintola A. Ige, Nwachukwu E. William, Ehimario Igumbor, Chinwe Ochu, Adesuyi A. Omoare, Olumide Okunoye, Stacie M. Greby, Molebogeng X. Rangaka, Andrew Copas, Ibrahim Dalhatu, Ibrahim Abubakar, Stephen McCracken, Matthias Alagi, Nwando Mba, Ahumibe Anthony, McPaul Okoye, Catherine Okoi, Oliver C. Ezechi, Babatunde L. Salako, Chikwe Ihekweazu, for the Nigeria SARS-CoV-2 Survey Group

**Affiliations:** 1 Nigerian Institute of Medical Research, Lagos, Nigeria; 2 Center for International Health, Education and Biosecurity, Institute of Human Virology, University of Maryland School of Medicine, Baltimore, Maryland, United States of America; 3 Malaria Branch, Division of Parasitic Diseases and Malaria, Center for Global Health, United States Centers for Disease Control and Prevention, Atlanta, Georgia, United States of America; 4 Division of Global HIV/AIDS and Tuberculosis, Center for Global Health, United States Centers for Disease Control and Prevention, Abuja, Nigeria; 5 Nigeria Centre for Disease Control, Abuja, Nigeria; 6 Institute for Global Health, University College London, London, United Kingdom; 7 Center for International Health, Education and Biosecurity, University of Maryland, Baltimore, Abuja, Nigeria; 8 School of Public Health, University of Western Cape, Cape Town, South Africa; 9 Division of Global HIV/AIDS and Tuberculosis, Center for Global Health, United States Centers for Disease Control and Prevention, Atlanta, Georgia, United States of America; National Institute of Allergy and Infectious Diseases, UNITED STATES

## Abstract

The observed epidemiology of SARS-CoV-2 in sub-Saharan Africa has varied greatly from that in Europe and the United States, with much lower reported incidence. Population-based studies are needed to estimate true cumulative incidence of SARS-CoV-2 to inform public health interventions. This study estimated SARS-CoV-2 seroprevalence in four selected states in Nigeria in October 2020. We implemented a two-stage cluster sample household survey in four Nigerian states (Enugu, Gombe, Lagos, and Nasarawa) to estimate age-stratified prevalence of SARS-CoV-2 antibodies. All individuals in sampled households were eligible for interview, blood draw, and nasal/oropharyngeal swab collection. We additionally tested participants for current/recent malaria infection. Seroprevalence estimates were calculated accounting for the complex survey design. Across all four states, 10,629 (96·5%) of 11,015 interviewed individuals provided blood samples. The seroprevalence of SARS-CoV-2 antibodies was 25·2% (95% CI 21·8–28·6) in Enugu State, 9·3% (95% CI 7·0–11·5) in Gombe State, 23·3% (95% CI 20·5–26·4) in Lagos State, and 18·0% (95% CI 14·4–21·6) in Nasarawa State. Prevalence of current/recent malaria infection ranged from 2·8% in Lagos to 45·8% in Gombe and was not significantly related to SARS-CoV-2 seroprevalence. The prevalence of active SARS-CoV-2 infection in the four states during the survey period was 0·2% (95% CI 0·1–0·4). Approximately eight months after the first reported COVID-19 case in Nigeria, seroprevalence indicated infection levels 194 times higher than the 24,198 officially reported COVID-19 cases across the four states; however, most of the population remained susceptible to COVID-19 in October 2020.

## Introduction

In Nigeria, the first identified case of COVID-19, caused by the novel severe acute respiratory syndrome coronavirus 2 (SARS-CoV-2), was announced on February 27, 2020 [1]. The Government of Nigeria suspended international air travel into the country on March 23, 2020 and recommended a nationwide lockdown on March 30, 2020; however, the lockdown was implemented differently on a state-by-state basis [2], and most lockdown measures were eased by late May 2020 [3]. By August 2020, 53,865 confirmed cases of COVID-19 and 1,013 deaths had been reported in the country [4]. However, these official statistics likely underestimated the true cumulative incidence of SARS-CoV-2 during the first six months of the epidemic in Nigeria.

Despite rapid scale-up of the molecular laboratory network across the country, Nigeria lagged behind other countries’ COVID-19 testing rates. In July 2020, Nigeria had conducted 834 COVID-19 tests per million population with a daily average testing rate of 0·02 per 1,000 people compared to 34,678 and 0·31, respectively, in South Africa and 10,388 and 0·04 in Ghana, respectively [5]. Low testing rates were initially driven by difficulties in procuring sufficient reagents, few testing laboratories in the country, and testing hesitancy due to stigma around COVID-19 as well as testing guidelines which targeted individuals with symptoms consistent with COVID-19.

The initial low testing rate and targeted testing strategy likely contributed to the true rate of SARS-CoV-2 infection being under-reported in Nigeria. Few rigorously conducted population-based studies on the true cumulative incidence of SARS-CoV-2 infection have been published from sub-Saharan Africa. A recent systematic review found that of population-level SARS-CoV-2 serosurveys published by the end of 2020, only 1% were from sub-Saharan Africa [6]. Several serological studies conducted early in the epidemic have shown higher infection rates in sub-Saharan Africa compared to COVID-19 case data. A regional study in Zambia [7] estimated a prevalence of previous and active infection of 10.6% of the population, 92 times higher than official reported cases by July 2020. A study in Cameroon estimated a 32% seroprevalence by the end of August 2020 [8], 444 times higher than the total reported cases at that time. Additional studies reported wide-ranging estimates of antibody-confirmed infection with SARS-CoV-2, including in Addis Ababa, Ethiopia (8.8% among community members in April 2020 [9] and 3.0% among asymptomatic persons recruited from clinical sites in May of 2020 [10]), in Kenya (9·1% nationwide and 22·7% in Nairobi in September 2020 [11]), in Blantyre, Malawi (12·3% among healthcare workers in May–June, 2020 [12]), and in South Africa (30·6%–46·2% among community members across Cape Town subdistricts in July–August 2020 [13]). In Nigeria, a small convenience sample of different venues in Niger State conducted in June 2020 found a seroprevalence of 25.4% [14]. Another study found 45.1% seroprevalence among a small sample of health workers in Ibadan, Nigeria [15], while seroprevalence of 17.4% (range 5.4% - 31.9%) was reported in surveys conducted in December 2020 in the 21 Local Government Areas of Anambra State [16].

The observed epidemiology of the SARS-CoV-2 epidemic in sub-Saharan Africa has varied greatly from the epidemic in high-income countries across Europe and the United States, most markedly in terms of much lower reported cases relative to population [17]. Representative population-based studies are needed to understand differences in the epidemiology of SARS-CoV-2 by region as well as estimate the true cumulative incidence of SARS-CoV-2 infection to inform public health intervention and response. This study estimated SARS-CoV-2 seroprevalence in the population of four selected states in Nigeria in October of 2020, using an adapted WHO Unity Study protocol for population-based household surveys [18]. The objectives of this study were to measure the prevalence of antibodies to SARS-CoV-2 in the populations of selected states in Nigeria by sex and age group. Additionally, given the non-specific symptomatology of COVID-19, which could hinder accurate diagnosis in settings where other endemic diseases such as malaria present similarly [19] as well as the need for information on the dynamics between malaria and SARS-CoV-2, we tested participants for current/recent malaria infection.

## Methods

### Study design and study population

We implemented a two-stage cluster sample cross-sectional survey of households in four states in Nigeria (Enugu, Gombe, Lagos, and Nasarawa) representing four of the six geopolitical zones of Nigeria (South-East, North-East, South-West, and North-Central, respectively) in October of 2020 (S1 Fig). The sampling frame consisted of 32,744 enumeration areas (EAs) and an estimated 23,191,138 individuals based on projections from the 2006 Census. The EAs from the 2006 Census used were mutually exclusive (non-overlapping) to ensure that within each stratum all households and residents had an equal chance of being included in the survey.

According to urban/rural categorizations of EAs by the National Population Commission of Nigeria, Enugu is 70%, Gombe is 23%; Lagos is 80%, and Nasarawa is 24% urban, respectively. Since several previous studies have indicated higher seroprevalence in urban compared to rural areas [20, 21], urban areas in Gombe and Nasarawa were oversampled in a 40:60 urban: rural ratio to provide a more precise estimate of seroprevalence in urban areas of these states. EAs in Lagos were sampled based on an 80:20 urban: rural ratio and proportionally to the number of EAs within each local government area. EAs in Enugu were sampled using probability proportional to estimated population size. Thirty EAs in Lagos and thirty-four EAs in the other three states were sampled. Teams mapped and listed all households in each sampled EA in Enugu, Gombe, and Nasarawa. In Lagos, due to time and resource limitations, 50–100 households were mapped per EA. During the second stage of sampling, 20 households per EA were selected for the survey through simple random sampling.

### Sample size

The overall sample size was determined by the number of blood draws needed to obtain relatively robust estimates (relative standard error (RSE) <0.4) of SARS-CoV-2 seroprevalence for each state. Assuming a 68% response rate [22], calculated as the household response rate multiplied by the individual response rate multiplied by the blood draw response rate, a total of 2,521 participating individuals in Gombe would be needed to achieve a RSE of 0.389 to detect a state-level prevalence of 1.24% (assuming 2% in urban areas and 1% in rural areas) with a 95% confidence interval (CI) of 0.26% to 2.22%; similar or fewer individuals would be needed in Enugu (n = 2,057) and Nasarawa (n = 2,421). All individuals of any age who were present in the house at the time of the survey were eligible for participation.

### Field procedures

Community leaders and members residing in sampled EAs were sensitized to the survey by mobilization teams recruited from within the community for two weeks prior to survey team entry into the EA. At sampled households, after obtaining informed consent, surveyors asked the head of household or other adult member who normally resided there to list all members of the household as well as answer a brief household-level questionnaire. Consenting household members were asked about their symptom history since March 2020 (to estimate the potential proportion of SARS-CoV-2 infections that were asymptomatic), testing and care-seeking for COVID-19, behaviors related to contact with a known SARS-CoV-2 case, travel within and outside of the country, transportation, attending markets, or going to work or school. Following the individual questionnaire, a venous blood sample of 6 mL was obtained from all consenting participants age 2–17 years and 10 mL for all consenting participants age 18 years and older using Ethylene Diamine Tetra-acetic Acid (EDTA)-containing vacuum tubes. One mL of capillary blood (finger or heel prick) was obtained from participants younger than age two years. From sampled blood, a malaria rapid diagnostic test (RDT) (SD Bioline Malaria Antigen Pf (HRP2)) was administered; participants testing positive for malaria were given antimalarial treatment per National Malaria Elimination Programme guidelines. Consenting individuals also had nasal and oropharyngeal (N/OP) swabs taken for assessing current SARS-CoV-2 infection status.

All data were captured on tablets using the CSPro application in Enugu, Gombe, and Nasarawa. In Lagos, REDCap was used for household data capture. Encrypted household, individual, and specimen data were uploaded to a central server daily.

### Ethics and informed consent

Survey teams first sought household-level consent to participate from the head of household or other household member aged 18 years or older. Consenting heads of households signed their name on the electronic household consent form programmed into the CSPro application. Individual consent covered the individual questionnaire and sample collection (blood draw, N/OP swabs); separate consent was obtained for storage of samples for future use. Individual consent was sought from adults aged 18 years and older; parental permission sought for those age 0 to 17 years; and parental permission and individual assent was sought for those age 10 to 17 years. Individuals who agreed to participate had their provision of informed consent or assent documented by signing their name on the electronic consent form which was countersigned by the interviewer. A paper copy was left with participants. The study was approved by the National Health Research Ethics Committee, the Nigerian Institute of Medical Research (NIMR) Institutional Review Board (IRB), the University of Maryland, Baltimore IRB, and the United States Centers for Disease Control and Prevention (CDC) COVID-19 response Human Subjects Review.

### Laboratory methods

N/OP swabs from the same participant were immediately placed in the same viral transport tube containing 2–3 mL of viral transport medium and transported at 2–8°C to the National Reference Laboratory (NRL) in Abuja and NIMR in Lagos. Temperature monitoring devices were used to track temperature excursions throughout sample transport. Swabs were stored at 2–8°C prior to testing at the NRL and NIMR. Blood samples were centrifuged at a regional laboratory in each state within eight hours of sample collection to separate plasma from serum and plasma was aliquoted into cryovials prior to transport at -20°C in Crēdo Cube (Peli Biothermal MN USA) to the NRL and NIMR. Plasma specimens were stored at the Biorepository of the NRL and NIMR at -80°C until testing.

The qualitative detection of nucleic acids from SARS-CoV-2 in N/OP swabs was performed using the cobas 8800/6800 system (Roche Diagnostics GmbH, Mannheim, Germany), a real-time reverse-transcription polymerase chain reaction (RT-PCR) test with fully automated sample preparation (nucleic acid extraction and purification) capacity, PCR amplification, and detection capability. The cobas SARS-CoV-2 PCR platform has two specific targets: target 1 (ORF1ab), a non-structural region for SARS-CoV-2, and target 2 (E gene), a conserved region in the structural protein envelope E gene for pan-sabercovirus detection.

Due to the potential for serological assay cross-reactivity [23, 24], a two-stage testing process was initially used to classify a sample as positive for SARS-CoV-2 antibodies. The Abbott Architect Plus i1000sr Analyzer (Abbott, Illinois, USA) and SARS-CoV-2 IgG kit targeting the nucleocapsid protein (NCP) and the Euroimmun Anti-SARS-CoV-2 NCP ELISA (IgG) (Euroimmun Medizinische Labordiagnostika, Lübeck, Germany) were used to test for antibodies to SARS-CoV-2. Samples that tested positive on either the Euroimmun or Abbott assay were then tested using the Luminex xMAP SARS-CoV-2 Multiple-Antigen IgG Assay on the Luminex MAGPIX instrument. A ten percent random sample of specimens testing negative on both Euroimmun and Abbott were also tested on the Luminex xMAP SARS-CoV-2 Multi-Antigen IgG assay (Table A in S1 Text). All tests were performed according to the manufacturer’s guidelines.

Positive RT-PCR results were sent to State Surveillance Officers for case investigation and contact tracing. Negative results were also communicated to the State Surveillance Officer who then conveyed these to participants. All results were reported to the Nigeria Centre for Disease Control (NCDC) for entry into the national COVID-19 surveillance platform.

### Statistical analysis

In Enugu, Gombe, and Nasarawa, household and individual population weights were created accounting for sample selection probabilities and nonresponse. Household response rates were calculated using the American Association for Public Health Opinion Research Rate method version 4.1 as the number of complete and incomplete household interviews among all eligible households, plus those estimated to be eligible among those with unknown eligibility (households not located, not attempted, or unreachable [25]). Vacant and destroyed households, and household units with no eligible respondents (e.g., adult or emancipated minor) to consent for the household were considered ineligible and excluded from the calculation. Individual response rates were calculated as the number of individuals interviewed divided by the number of individuals eligible to participate. As all individuals in the household were eligible for inclusion in the survey, the individual analysis weight is the same for all members of the household. Blood draw, swab, and malaria response rates were calculated as the number of individuals providing a specimen divided by the number of individuals interviewed. As household and individual response rates were not tracked in Lagos, post-stratification weights accounting for age and sex were used to generalize to the Lagos State population. A wealth score was created for Enugu, Gombe, and Nasarawa combined and separately for Lagos State using principal component analysis and divided into quintiles [26].

Seropositivity to SARS-CoV-2 was defined as samples positive on either the Abbott or Euorimmun assay and on the Luminex xMAP SARS-CoV-2 Multi-Antigen IgG assay. Active SARS-CoV-2 infection was defined as a positive RT-PCR test. Descriptive analyses (e.g., proportions) and prevalence ratios (PR) of seropositivity from Poisson regression with robust standard errors were calculated accounting for the complex survey design and analysis weights. Variables significant at p<0.2 in bivariate analyses were included in a multivariable model and the Akaike information criterion (AIC) was used to select the best-fitting model [27]. Analyses were conducted using SAS 9.4 (Cary, NC) and Stata 16 (College Station, TX).

## Results

A total of 2,634 households were randomly sampled for inclusion and 2,509 participated in the survey. In Enugu, Gombe, and Nasarawa 652, 635, and 644 households were eligible for participation and 615 (94·8%), 605 (95·3%), and 607 (94·3%), respectively, agreed to participate. A total of 2,236 individuals in Enugu, 3,566 in Gombe, and 2,766 in Nasarawa were eligible to participate and 2,170 (97·0%), 3,488 (97·8%), and 2,698 (97·5%), respectively, agreed to be interviewed. A total of 2,659 people were interviewed in Lagos State. The blood draw response rates in Enugu, Gombe, Lagos, and Nasarawa were 98·9%, 98·4%, 90·0%, and 98·5%, respectively. Across all four states, a total of 11,015 individuals participated in the interview and 10,629 (96·5%) provided a blood sample.

Approximately half the survey respondents were male, most resided in urban areas (70·0%), and most (92·7%) reported no underlying health conditions, including pregnancy (Table 1). The weighted seroprevalence of SARS-CoV-2 antibodies was 25·2% (95% CI 21·8–28·6) in Enugu State, 9·3% (95% CI 7·0–11·5) in Gombe State, 23·3% (95% CI 20·5–26·4) in Lagos State, and 18·0% (95% CI 14·4–21·6) in Nasarawa State (Table 2). Households with 1–2 people had the lowest household seropositivity with only 30.2% with 1–2 seropositive members (Table B in S1 Text). The weighted prevalence of active SARS-CoV-2 infection during the survey period was 0·2% (95% CI 0·1–0·4). Across all age groups, a slightly higher proportion of SARS-CoV-2 antibody-negative participants reported at least one symptom consistent with COVID-19 than SARS-CoV-2 antibody-positive participants, but none of these differences was statistically significant (Table C in S1 Text).

**Table 1:** Weighted distribution of survey participant demographic characteristics (n = 11,015), Nigeria, October 2020.

Characteristic	Percent %	95% Confidence Interval
Sex		
Male	49.5	(47.7–51.3)
Female	50.6	(48.8–52.3)
Age, years		
0–4	11.7	(9.8–13.5)
5–9	14.6	(13.3–15.8)
10–14	12.0	(11.0–13.0)
15–19	8.4	(7.7–9.1)
20–29	13.8	(12.4–15.3)
30–39	8.6	(7.9–9.3)
40–49	12.2	(10.9–13.5)
50–59	7.2	(6.3–8.2)
60 or older	11.5	(9.8–13.1)
Location		
Rural	30.0	(22.1–37.9)
Urban	70.0	(62.1–77.9)
Health conditions		
Currently pregnant*	2.4	(1.6–3.3)
Diabetes	1.0	(0.6–1.3)
Hypertension	3.2	(2.5–3.8)
Heart Disease	0.6	(0.3–0.9)
Asthma	0.3	(0.2–0.5)
Tuberculosis	0.0	(0.0–0.1)
Chronic kidney disease	0.2	(0.1–0.3)
Emphysema/chronic bronchitis/COPD**	0.1	(0.0–0.2)
HIV	0.1	(0.1–0.2)
Sickle cell disease	0.2	(0.1–0.3)
Cancer	0.1	(0.00–0.2)
None reported	92.7	(91.7–94.1)

*Denominator restricted to women age 15 to 49 years.

**Chronic obstructive pulmonary disease.

**Table 2:** Prevalence of SARS-CoV-2 antibodies among survey participants by state and demographic characteristics, Nigeria, October 2020 (n = 10,629).

	Enugu (n = 2,147)		Gombe(n = 3,432)		Nasarawa (n = 2,657)		Lagos (n = 2,393)	
	Percent	95% CI	Percent	95% CI	Percent	95% CI	Percent	95% CI
**Sex**								
Male	23.9	20.4–27.3	11.0	8.2–13.8	19.3	14.9–23.8	23.7	20.3–27.4
Female	26.3	22.0–30.6	7.5	5.3–9.7	16.8	13.4–20.2	23.0	19.7–26.6
**Residence**								
Rural	20.2	15.1–25.3	9.4	6.4–12.3	15.8	11.3–20.3	19.2	14.3–25.2
Urban	28.2	25.2–31.2	9.0	5.8–12.2	22.9	19.5–26.3	24.0	20.9–27.4
**Age, years**								
Less than 5	15.1	10.6–19.7	5.9	2.4–9.3	6.9	2.2–11.6	17.7	11.3–26.6
5–9	19.0	13.8–24.2	5.1	3.0–7.2	15.1	9.9–20.3	20.0	16.0–27.1
10–14	27.5	21.9–33.2	7.8	4.4–11.3	18.3	12.9–23.7	28.2	23.1–33.8
15–19	33.9	23.3–44.6	14.4	10.0–18.7	22.1	14.9–29.2	28.6	23.6–34.2
20–29	28.4	21.7–35.2	9.4	5.8–13.0	16.0	11.9–20.1	23.2	19.5–27.2
30–39	30.4	24.5–36.3	12.2	7.5–16.9	25.8	18.2–33.4	20.8	16.4–26.0
40–49	24.5	17.3–31.8	13.1	7.5–18.6	26.3	18.9–33.6	24.0	19.1–29.7
50–59	30.5	23.2–37.9	16.8	10.8–22.8	15.3	7.3–23.3	23.7	16.1–33.4
60 or older	23.8	16.7–30.8	10.9	5.4–16.5	23.3	15.2–31.4	23.4	17.9–26.4
**Total**	**25.2**	**21.8–28.6**	**9.3**	**7.0–11.5**	**18.0**	**14.4–21.6**	**23.3**	**20.5–26.4**

* CI = confidence interval.

The estimated cumulative number of SARS-CoV-2 infections across the four states at the end of October 2020 based on seroprevalence estimates from the survey was 4,691,977; 194 times the cumulative number of cases officially reported across the four states (Table 3). The ratio of reported to estimated cumulative incidence was 1:728 in Enugu, 1:134 in Gombe, 1:135 in Lagos, and 1:1,211 in Nasarawa.

**Table 3:** Estimated number of cumulative SARS-CoV-2 infections in four states in Nigeria (October 2020).

	Enugu	Gombe	Lagos	Nasarawa
2018 census population projection*, n	4,375,683	1,380,672	12,348,505	3,242,784
Estimated number of SARS-CoV-2 infections, n (95% CI*)	1,102,672 (935,899–1,251,445)	128,402 (96,647–158,777)	2,877,202 (2,531,444–3,260,005)	583,701 (466,961–700,441)
Reported cases in official statistics^†^, n	1,514	957	21,245	482
Ratio of reported cases to estimated cumulative cases	1:728	1:134	1:135	1:1,211

*Population projections from the 2006 Census, National Population Council, Nigeria.

^†^Nigeria Centre for Disease Control, as of October 31, 2020.

*CI = Confidence interval.

The overall prevalence of malaria by HRP2-based RDT was 22.8% in Enugu, 45.8% in Gombe, 40.4% in Nasarawa, and 2.8% in Lagos and was not related to SARS-CoV-2 seroprevalence (Tables D and E in S1 Text). Malaria was more common among rural versus urban respondents: 31.8% versus 6.8% for malaria alone, and 4·4% versus 1·8% for malaria with SARS-CoV-2 antibodies.

**Fig 1:**
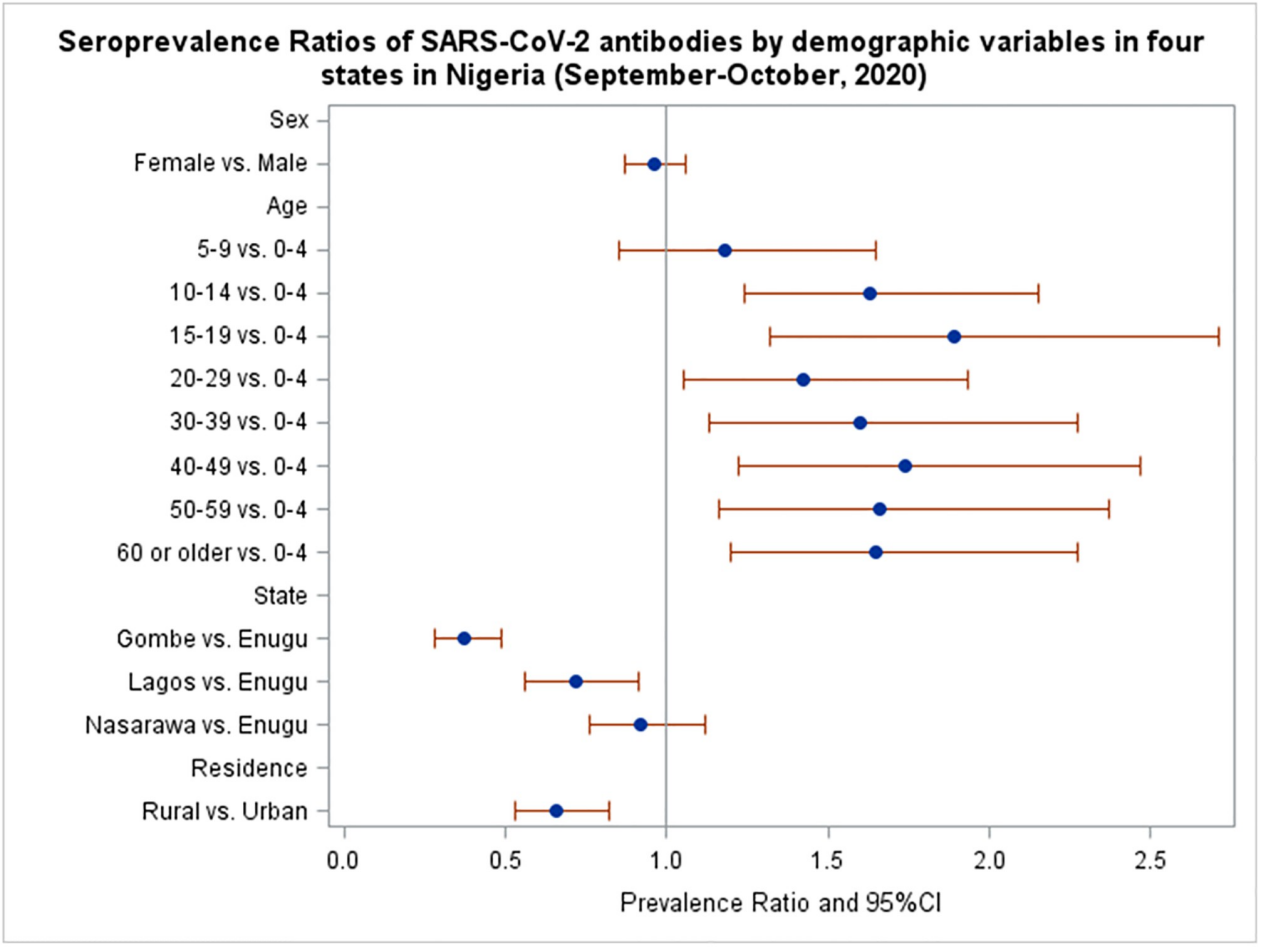
Unadjusted seroprevalence ratios for the presence of SARS-CoV-2 antibodies by demographic variables from four states in Nigeria (October 2020).

The prevalence of SARS-CoV-2 antibodies did not differ by sex (PR for women versus men = 0·96; 95% CI 0·86–1·06) (Table 4). All age groups 10 years and older had significantly higher prevalence of SARS-CoV-2 antibodies compared to children age 0 to 4 years (Table 4, Fig 1). Survey respondents from Gombe and Nasarawa States had significantly lower seroprevalence of SARS-CoV-2 antibodies compared to residents of Enugu State. Residents of rural areas had lower seroprevalence than those from urban EAs (PR 0·66, 95% CI 0·53–0·82), although this was no longer significant when adjusted for state and other factors, including age, residence, wealth, asthma, HIV, direct contact with a symptomatic person/confirmed case, and visit to the market (aPR = 0·83, 95% CI: 0.66–1.04). Adjusted for other factors, age 10 years or greater was associated with a higher seroprevalence of SARS-CoV-2 compared to age 9 and under. Asthma was also associated with a higher prevalence of SARS-CoV-2 antibodies after adjusting for other factors (aPR 1·99, 95% CI 1·13–3·51). Higher frequency of weekly market visits was associated with SARS-CoV-2 seropositivity in univariable analysis but this was not statistically significant when adjusted for other covariates (compared to no visits: aPR 1–2 visits 1.10, 95% CI 0.93–1.31; 3–5 visits 1.12, 95% CI 0.89–1.40; 6 or more 1.29, 95% CI 0.99–1.69) (Table 4).

**Table 4:** Associations between presence of SARS-CoV-2 antibodies and demographic and behavioral risk factors in four states in Nigeria in October 2020 (n = 9,296).

			Unadjusted			Adjusted		
Characteristics	Prevalence, %	95% CI	Prevalence Ratio^a^	95%CI	p-value	Prevalence Ratio	95%CI	p-value
**Sex**	.							
Male	21.2	(18.9–23.7)	1.0					
Female	20.3	(18.1–22.6)	1.0	(0.9–1.1)	0.405			
**Age, years**	.							
0–4	13.9	(9.6–18.2)	1.0			1.0		
5–9	16.4	(13.1–19.7)	1.2	(0.8–1.7)	0.330	1.2	(0.9–1.6)	0.190
10–14	22.5	(19.4–25.7)	1.6	(1.2–2.1)	0.001	1.6	(1.2–2.2)	0.001
15–19	26.1	(22.6–29.7)	1.9	(1.3–2.7)	0.001	2.0	(1.4–2.8)	0.000
20–29	19.7	(16.8–22.7)	1.4	(1.1–1.9)	0.023	1.6	(1.1–2.2)	0.008
30–39	22.1	(19.0–25.4)	1.6	(1.1–2.3)	0.008	1.8	(1.3–2.5)	0.000
40–49	24.0	(20.4–27.7)	1.7	(1.2–2.5)	0.002	1.7	(1.2–2.4)	0.007
50–59	23.0	(17.0–29.0)	1.7	(1.2–2.4)	0.006	1.6	(1.0–2.4)	0.035
>60	22.9	(18.8–27.1)	1.7	(1.2–2.3)	0.002	1.6	(1.2–2.3)	0.006
**State**	.							
Enugu	25.2	(21.8–28.7)	1.0			1.0		
Gombe	9.3	(7.0–11.6)	0.4	(0.3–0.5)	0.000	0.4	(0.3–0.6)	0.000
Nasarawa	18.0	(14.5–21.7)	0.7	(0.6–0.9)	0.007	0.8	(0.6–1.0)	0.042
Lagos	23.2	(20.1–26.5)	0.9	(0.8–1.1)	0.407	0.9	(0.7–1.2)	0.439
**Location**	.							
Urban	23.1	(20.4–25.9)	1.0			1.0		
Rural	15.2	(12.6–17.9)	0.7	(0.5–0.8)	0.000	0.8	(0.7–1.0)	0.113
**Education**	.							
No education	18.8	(15.9–21.9)	1.0					
Some primary	22.0	(17.1–27.1)	1.2	(0.9–1.5)	0.244			
Completed primary	25.1	(20.2–30.1)	1.3	(1.1–1.7)	0.012			
Some secondary	26.0	(22.3–29.8)	1.4	(1.1–1.7)	0.002			
Completed secondary or higher	19.9	(17.4–22.6)	1.1	(0.9–1.3)	0.563			
**Wealth quintile**	.							
Lowest	16.4	(13.1–19.8)	1.0			1.0		
Second	16.7	(13.7–19.7)	1.0	(0.8–1.3)	0.901	0.8	(0.7–1.1)	0.163
Middle	24.1	(20.3–28.1)	1.5	(1.2–1.9)	0.001	1.1	(0.9–1.4)	0.321
Fourth	22.1	(18.2–26.0)	1.4	(1.1–1.7)	0.022	1.1	(0.8–1.4)	0.593
Highest	24.1	(21.8–26.5)	1.5	(1.2–1.8)	0.000	1.2	(0.9–1.4)	0.191
**Household size**								
1–2	22.8	(18.5–27.0)	1.0					
3–5	21.8	(19.0–24.5)	1.0	(0.8–1.1)	0.607			
6–9	19.6	(17.2–22.1)	0.9	(0.7–1.0)	0.109			
≥10	15.2	(11.1–19.2)	0.7	(0.5–0.9)	0.014			
**Any comorbid condition**	.							
No	21.0	(19.0–23.1)	1.0					
Yes	20.5	(16.5–24.4)	1.0	(0.8–1.2)	0.766			
**Cardiovascular disease**	.							
No	20.9	(18.9–23.0)	1.0					
Yes	18.8	(6.1–31.6)	0.4	(0.5–1.7)	0.746			
**Diabetes**	.							
No	20.9	(18.9–23.0)	1.0					
Yes	22.5	(13.6–31.5)	1.1	(0.7–1.6)	0.709			
**Chronic Kidney Disease**	.							
No	20.9	(18.9–23.0)	1.0					
Yes	20.4	(4.0–36.8)	1.0	(0.4–2.1)	0.945			
**Hypertension**	.							
No	20.9	(18.8–23.0)	1.0					
Yes	22.9	(17.4–28.6)	1.1	(0.9–1.4)	0.447			
**Asthma**	.							
No	20.8	(18.8–22.9)	1.0			1.0		
Yes	43.4	(16.0–70.9)	2.1	(1.1–3.9)	0.022	2.0	(1.1–3.5)	0.018
**Pregnant**	.							
No	23.4	(20.6–26.3)	1.0					
Yes	16.7	(8.0–25.5)	0.7	(0.4–1.2)	0.218			
**HIV**	.							
No	21.0	(18.9–23.1)	1.0			1.0		
Yes	7.9	(0.0–18.7)	0.4	(0.1–1.5)	0.157	0.5	(0.1–1.8)	0.260
**Malaria**	.							
No	21.7	(19.5–24)	1.0					
Yes	15.6	(12.9–18.3)	0.7	(0.6–0.9)	0.000			
**Direct contact with someone with any symptom or confirmed COVID**	.							
No	20.7	(18.7–22.8)	1.0			1.0		
Yes	27.8	(18.9–36.8)	1.3	(1.0–1.9)	0.070	1.3	(1.0–1.8)	0.076
**Traveled within Nigeria since March 2020**	.							
No	20.9	(18.8–23.0)	1.0					
Yes	21.8	(16.9–26.7)	1.0	(0.8–1.3)	0.711			
**Number weekly visits to the market**	.							
0	16.5	(14.0–19.0)	1.0			1.0		
1–2	22.9	(19.8–26.0)	1.4	(1.2–1.7)	0.000	1.1	(0.9–1.3)	0.255
3–5	24.8	(19.8–29.9)	1.5	(1.2–1.9)	0.001	1.1	(0.9–1.4)	0.337
6 or more	27.3	(22.8–32.0)	1.7	(1.3–2.1)	0.000	1.3	(1.0–1.7)	0.064
**Attended gatherings of more than 20 people**	.							
No	20.6	(18.2–23.1)	1.0					
Yes	21.4	(18.8–24.1)	1.0	(0.9–1.2)	0.583			
**Frequency of using public transport per week**	.							
0	20.1	(16.9–23.4)	1.0					
1–2 times	24.2	(21.9–26.6)	1.2	(1.0–1.4)	0.022			
3–4 times	24.2	(19.0–29.4)	1.2	(0.9–1.5)	0.136			
> = 5 times	22.5	(18.1–27)	1.1	(0.9–1.36)	0.265			
**Transport, Taxi**	.							
No	23.5	(20.7–26.3)	1.0					
Yes	27.0	(20.8–33.3)	1.2	(0.9–1.5)	0.279			
**Transport, Motorbike**	.							
No	24.1	(21.0–27.4)	1.0					
Yes	23.4	(20.4–26.6)	1.0	(0.8–1.1)	0.672			
**Transport, Keke/napep**	.							
No	23.0	(20.3–25.9)	1.0					
Yes	24.6	(20.7–28.5)	1.1	(0.9–1.3)	0.475			
**Transport, Bus/minibus**	.							
No	22.4	(19.5–25.5)	1.0					
Yes	25.4	(21.7–29.3)	1.1	(1.0–1.4)	0.163			
**Transport, Other**	.							
No	23.2	(20.6–26.0)	1.0					
Yes	31.1	(20.7–41.6)	1.3	(0.9–1.9)	0.101			

^a^The prevalence ratios reflect the prevalence in once stratum compared to the reference group of the category.

Note: Positive responses for travel outside Nigeria and report of previous COVID-19 among participants were too few to include in models.

## Conclusions

The seroprevalence of SARS-CoV-2 antibodies was much higher than expected, and the prevalence of active SARS-CoV-2 infection was low in this population-based seroprevalence survey from four states covering four of the six geopolitical zones of Nigeria. The active SARS-CoV-2 infection finding was consistent with low reported community transmission at the time the study was implemented, which coincided with the trough between the first and second waves of the epidemic in Nigeria [28]. The higher-than-expected seroprevalence of antibodies eight months into the epidemic in Nigeria may indicate that SARS-CoV-2 was circulating in Nigeria earlier than the first detected imported case suggests [29] and that there was much more widespread transmission of SARS-CoV-2 than official statistics would indicate, despite swift containment measures.

By October 31, 2020 according to official statistics, a total of 62,944 SARS-CoV-2 infections had been detected in Nigeria [1], with reported infection rates per 100,000 population of 35 in Enugu, 69 in Gombe, 172 in Lagos, and 15 in Nasarawa; approximately 130 to 1,200 times lower than cumulative infection rates at the time depending on the state. This study found no significant differences in the SARS-CoV-2 seroprevalence between Enugu and Lagos, and similar seroprevalence in Nasarawa; all three states had had significantly higher seroprevalence than Gombe. This underscores the importance of population-based seroprevalence estimates that are not subject to bias from unequal distribution or uptake of testing services during outbreaks for development of accurate public health mitigation measures. Even in sub-Saharan countries with much higher COVID-19 testing rates, many infections are not captured: one pre-print study in South Africa found that 95% of infections were not reported to the national surveillance system [30].

We found significantly higher seroprevalence in those age 10 years or older compared to younger participants in this population-based survey. The relationship between age and SARS-CoV-2 infection is varied across settings, but several other studies have found also lower seroprevalence among younger children [31, 32]. Other risk factors for SARS-CoV-2 infection in these Nigerian states included asthma, although this finding should be interpreted with caution due to the low absolute frequency of asthma (n = 10 people in total with asthma across the four states). Frequent visits to the market and reported contact with someone with COVID-19/symptoms suggestive of COVID-19 showed some association with the prevalence of SARS-CoV-2 antibodies.

The need for valid seroprevalence measures during active outbreaks of pathogens with predominantly asymptomatic phenotypes is further emphasized by our findings of similar, if not lower, reporting of symptoms among SARS-CoV-2 antibody-positive respondents in our survey compared to those testing negative for antibodies. The observed similarity in symptom reporting by seroprevalence status was consistent across all four states. Symptom based testing strategies, while justified based on test kit availability and laboratory capacity, significantly underestimated the burden of SARS-CoV-2 in Nigeria. It is important to note that the relationship between detected antibodies and immunity to subsequent SARS-Cov-2 infections is still unclear; one longitudinal study in Wuhan, China, found that only about 40 percent of those with asymptomatic SARS-CoV-2-infections had neutralizing antibodies, although that proportion remained relatively stable over six–seven months [33].

Despite the robust sampling approach, this study had several key limitations. People with mild or asymptomatic SARS-CoV-2 infections have been shown to mount a less robust antibody response (than those with moderate or severe infections), and antibody responses might decline more rapidly over time to undetectable levels [34–36]. Thus, it is possible that our findings underestimate seroprevalence in these states, especially given the low levels of symptoms reported. Despite some of these inherent limitations of SARS-CoV-2 serological assays, especially in asymptomatic populations, we used a three-test algorithm to improve specificity, given documented issues of cross-reactivity of SARS-CoV-2 serological assays in malaria-endemic settings [23, 37]; all assays were validated in Nigeria [38], with the best-performing assay used to determine seroprevalence.

This serosurvey provides population-based estimates of SARS-CoV-2 infections in four states in Nigeria after the first wave of COVID-19. Approximately eight months after the first COVID-19 case in Nigeria, seroprevalence ranged from 9.3% in Gombe to 25.2% in Enugu. Although seroprevalence indicated infections that were up to 1,000 times that of officially reported COVID-19 cases, the vast majority of the population in Nigeria remained susceptible to COVID-19 infection as of October 2020.

## Supporting information

S1 Fig Time period serosurvey implementation in the context of the epidemic curve for Nigeria with seven-day rolling case average. The green line represents the 7-day rolling average of daily new confirmed COVID-19 cases per million people. The gray box represents the period during which samples were collected during the survey. Source: Johns Hopkins University CSSE COVID-19 data.
(TIFF)

S1 Text (DOCX)

S1 Acknowledgments (DOCX)
