# Supplementary figures and images for: Seroprevalence of SARS-CoV-2 in four states of Nigeria in October 2020: A population-based household survey
Source: PLOS Glob Public Health. 2022 Jun 17;2(6):e0000363. doi: 10.1371/journal.pgph.0000363 (PMC10022353; doi:10.1371/journal.pgph.0000363)

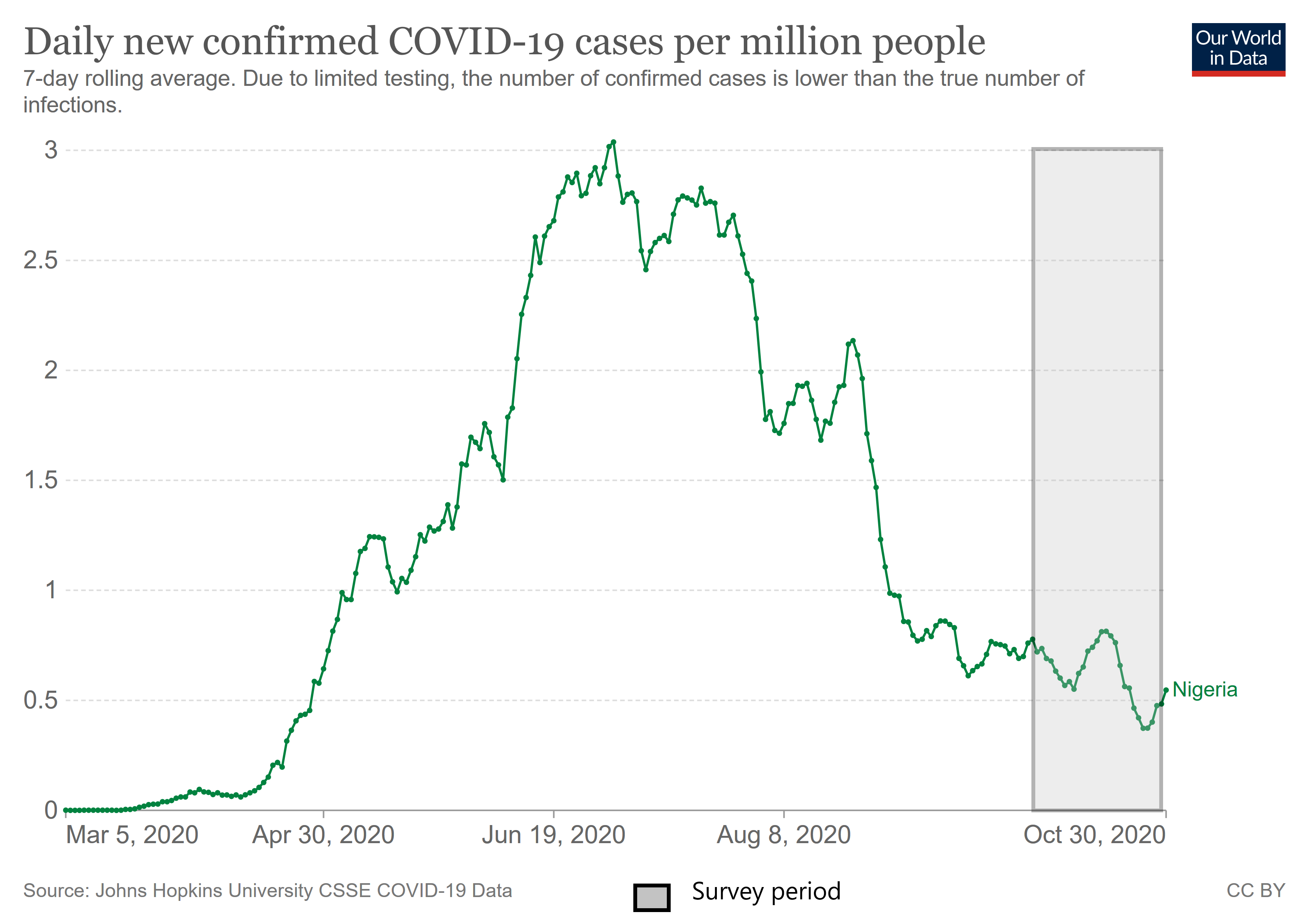

Supplement: S1 Fig — The green line represents the 7-day rolling average of daily new confirmed COVID-19 cases per million people. The gray box represents the period during which samples were collected during the survey. Source: Johns Hopkins University CSSE COVID-19 data. (TIFF) [file pgph.0000363.s001.tiff]
