# Supplementary material for: Seroprevalence of SARS-CoV-2 in four states of Nigeria in October 2020: A population-based household survey
Source: PLOS Glob Public Health. 2022 Jun 17;2(6):e0000363. doi: 10.1371/journal.pgph.0000363 (PMC10022353; doi:10.1371/journal.pgph.0000363)
Supplement: S1 Text — (DOCX) [file pgph.0000363.s002.docx]

Table A. Results of samples tested by Luminex xMAP SARS-CoV-2 Multi-Antigen IgG assay, based on results on Abbott or Euorimmun assays.

|  |  |  | **Total tested on Luminex** | **Luminex result** | |
| --- | --- | --- | --- | --- | --- |
| **Abbott** | **Euroimmun** | **Total** |  | **Negative** | **Positive** |
| Negative | Negative | 6,316 | 501 | 474 (94.6) | 27 (5.4) |
| Positive | Negative | 242 | 242 | 148 (61.2) | 94 (38.8) |
| Negative | Positive | 2,018 | 2,012 | 1,311 (65.2)* | 701 (34.8) |
| Positive | Positive | 1,565 | 1,563 | 423 (27.1)* | 1,140 (72.9) |
| * Differences between Total tested on Luminex and Luminex Negative and Positive are due to "No Call" results. | | | | | |

Table B: Distribution of households by the number of seropositive individuals and household size

| **Household size** | **Number of seropositive individuals at household** | | | | | | | |
| --- | --- | --- | --- | --- | --- | --- | --- | --- |
|  | **None** | | **1-2** | | **3-5** | | **6+** | |
|  | **n** | **%*** | **n** | **%** | **n** | **%** | **n** | **%** |
| 1-2 | 427 | 69.8 | 163 | 30.2 | 0 |  | 0 |  |
| 3-5 | 565 | 50.1 | 452 | 43.0 | 68 | 6.9 | 0 |  |
| 6-9 | 239 | 33.5 | 239 | 50.9 | 84 | 15.0 | 6 | 1.0 |
| 10+ | 30 | 31.3 | 38 | 37.8 | 26 | 24.1 | 6 | 6.8 |
| *Weighted percentages | | |  |  |  |  |  |  |

Table C.Reported symptoms by SARS-CoV-2 antibody status and age group among survey participant from four states in Nigeria, October 2020 (n = 10,628)

|  | **Antibody Positive (n=1937)** | | | | | | | | | **Antibody Negative (n=8691)** | | | | | | | | |
| --- | --- | --- | --- | --- | --- | --- | --- | --- | --- | --- | --- | --- | --- | --- | --- | --- | --- | --- |
|  | **Children* (n=331)** | | **Adolescents and young adults***  **(n=412)** | | **Adults***  **(n=1072)** | | **Senior Adults* (n=122)** | | **Children* (n=2447)** | | **Adolescents and young adults***  **(n=1719)** | | **Adults***  **(n=4047)** | | **Senior Adults* (n=478)** | | |  |
| **Symptoms since March 2020** | **n** | **% ^α^** | **n** | **%** | **n** | **%** | **n** | **%** | **n** | **%** | **n** | **%** | **n** | **%** | **n** | | **%** |  |
| Fever | 68 | 10.8 | 83 | 11.1 | 233 | 15.4 | 31 | 12.5 | 494 | 14.7 | 310 | 13.8 | 885 | 16.5 | 107 | | 12.4 |  |
| Rash | 7 | 1.7 | 6 | 1.4 | 16 | 0.8 | 1 | 0.4 | 57 | 1.2 | 30 | 1.5 | 73 | 1.0 | 12 | | 2.3 |  |
| Chills | 19 | 2.8 | 27 | 4.5 | 75 | 4.0 | 8 | 2.6 | 133 | 3.7 | 120 | 4.6 | 250 | 3.6 | 51 | | 4.6 |  |
| Diarrhea | 14 | 1.9 | 12 | 2.4 | 36 | 1.9 | 8 | 2.7 | 117 | 2.9 | 44 | 1.5 | 98 | 1.4 | 25 | | 2.3 |  |
| Cough | 41 | 4.9 | 51 | 6.0 | 103 | 5.2 | 15 | 7.5 | 306 | 9.8 | 172 | 6.9 | 394 | 6.9 | 83 | | 8.9 |  |
| Vomiting | 20 | 2.4 | 18 | 2.6 | 18 | 0.8 | 2 | 0.6 | 134 | 3.7 | 85 | 3.6 | 79 | 1.2 | 5 | | 0.3 |  |
| Sore throat | 0 | 0.0 | 2 | 0.9 | 19 | 1.9 | 0 | 0.0 | 5 | 0.1 | 18 | 0.5 | 50 | 1.1 | 8 | | 1.0 |  |
| Loss of appetite | 10 | 1.1 | 15 | 1.6 | 31 | 1.8 | 3 | 1.1 | 52 | 1.9 | 39 | 1.3 | 107 | 1.4 | 11 | | 1.4 |  |
| Shortness of breath | 2 | 0.2 | 1 | 0.7 | 13 | 0.7 | 1 | 0.4 | 2 | 0.0 | 5 | 0.2 | 32 | 0.6 | 14 | | 1.5 |  |
| Muscle aches | 0 | 0.0 | 1 | 0.2 | 24 | 2.5 | 6 | 3.4 | 1 | 0.0 | 6 | 0.5 | 80 | 2.5 | 27 | | 8.0 |  |
| Fatigue | 5 | 0.8 | 6 | 0.9 | 52 | 3.4 | 7 | 2.2 | 23 | 0.5 | 30 | 1.0 | 200 | 4.0 | 28 | | 4.0 |  |
| Conjunctivitis/pink eye | 1 | 0.2 | 1 | 0.2 | 4 | 0.1 | 1 | 0.3 | 10 | 0.2 | 11 | 0.6 | 28 | 0.6 | 5 | | 1.0 |  |
| Runny nose | 44 | 7.3 | 38 | 4.7 | 108 | 6.1 | 12 | 3.6 | 343 | 9.1 | 168 | 7.2 | 448 | 7.0 | 46 | | 4.1 |  |
| Joint aches | 2 | 0.3 | 7 | 0.8 | 61 | 3.9 | 22 | 11.2 | 21 | 0.8 | 30 | 1.5 | 243 | 5.6 | 79 | | 10.7 |  |
| Headache | 47 | 7.8 | 89 | 12.4 | 279 | 17.5 | 28 | 12.9 | 330 | 9.6 | 382 | 15.8 | 1044 | 18.5 | 116 | | 13.5 |  |
| Loss of smell (anosmia) | 0 | 0.0 | 2 | 0.2 | 10 | 0.6 | 2 | 0.8 | 1 | 0.0 | 4 | 0.1 | 47 | 0.8 | 6 | | 0.7 |  |
| Nosebleed | 0 | 0.0 | 0 | 0.0 | 3 | 0.1 | 0 | 0.0 | 4 | 0.1 | 5 | 0.2 | 5 | 0.1 | 1 | | 0.2 |  |
| Loss of taste (ageusia) | 0 | 0.0 | 2 | 0.3 | 5 | 0.2 | 2 | 2.1 | 5 | 0.2 | 3 | 0.1 | 20 | 0.3 | 6 | | 0.5 |  |
| Seizures | 0 | 0.0 | 0 | 0.0 | 0 | 0.0 | 1 | 0.3 | 2 | 0.0 | 2 | 0.1 | 4 | 0.2 | 0 | | 0.0 |  |
| Altered consciousness | 3 | 0.3 | 0 | 0.0 | 9 | 0.4 | 0 | 0.0 | 0 | 0.0 | 11 | 0.3 | 21 | 0.4 | 9 | | 0.8 |  |
| Other symptom | 1 | 0.2 | 0 | 0.0 | 7 | 0.5 | 3 | 1.9 | 9 | 0.3 | 7 | 0.4 | 35 | 0.8 | 10 | | 1.0 |  |
|  |  |  |  |  |  |  |  |  |  |  |  |  |  |  |  | | |  |
| **Symptomatic**** | 106 | 17.0 | 138 | 19.6 | 455 | 29.7 | 60 | 30.9 | 914 | 27.5 | 632 | 28.2 | 1748 | 34.2 | 230 | | 33.7 |  |
| *Children defined as less than age 10 years, adolescents and young adults defined as age 10-17 years, adults defined as age 18-64 years, and senior adults defined as age 65 years and over.  **Symptomatic individuals are defined as those reporting any symptom since March 2020.  ^α^ All percentages are weighted. | | | | | | | | | | | | | | | | |  |  |

Table D. Seroprevalence of SARS-CoV-2 antibodies by current/recent malaria infection, stratified by state, Nigeria, October 2020 (n=10,508)

| State | Malaria positive, % | SARS-CoV-2 antibodies, % |  | SARS-CoV-2 seroprevalence by current/recent malaria infection | | | | |
| --- | --- | --- | --- | --- | --- | --- | --- | --- |
|  |  |  |  | Prevalence, % | 95% CI | Prevalence Ratio | 95% CI | p-value |
| State |  |  | Malaria |  |  |  |  |  |
| Enugu | 22.82 | 25.21 | No | 24.42 | (20.56 - 28.29 ) | 1 |  |  |
|  |  |  | Yes | 27.93 | (23.65 - 32.21 ) | 1.14 | (0.94 - 1.40) | 0.184 |
| Gombe | 45.84 | 9.25 | No | 10.02 | (7.43 - 12.60 ) | 1 |  |  |
|  |  |  | Yes | 8.32 | (5.50 - 11.14 ) | 0.83 | (0.60 - 1.15) | 0.255 |
| Nasarawa | 40.42 | 18.03 | No | 18.90 | (15.22 - 22.57 ) | 1 |  |  |
|  |  |  | Yes | 16.34 | (11.66 - 21.02 ) | 0.86 | (0.67 - 1.11) | 0.241 |
| Lagos | 2.82 | 23.19 | No | 23.20 | (20.04 - 26.37 ) | 1 |  |  |
|  |  |  | Yes | 18.46 | (7.90 - 29.01 ) | 0.8 | (0.44 - 1.43) | 0.429 |

Table E. Estimated prevalence of SARS-CoV-2 and malaria co-infection among survey participants from four states in Nigeria October 2020 (n=10,508)

|  | SARS-CoV-2^β^ only (n=1463) | | Malaria only (n=2599) | | Co-infection (n=445) | | Not infected with either (n=6001) | |
| --- | --- | --- | --- | --- | --- | --- | --- | --- |
|  | **n** | **%^α^** | **n** | **%** | **n** | **%** | **n** | **%** |
| **Characteristics** |  |  |  |  |  |  |  |  |
| **Residence** |  |  |  |  |  |  |  |  |
| Urban | 1027 | 21.1 | 922 | 6.7 | 239 | 1.8 | 3769 | 70.3 |
| Rural | 436 | 10.8 | 1677 | 31.8 | 206 | 4.4 | 2232 | 53.1 |
| **Sex** |  |  |  |  |  |  |  |  |
| Male | 648 | 18.0 | 1350 | 14.6 | 252 | 3.1 | 2647 | 64.2 |
| Female | 815 | 18.1 | 1249 | 13.6 | 193 | 2.1 | 3354 | 66.1 |
| **Age groups*** |  |  |  |  |  |  |  |  |
| Children | 202 | 12.3 | 1084 | 23.6 | 125 | 2.9 | 1329 | 61.2 |
| Adolescents and young adults | 262 | 18.5 | 763 | 22.7 | 142 | 4.7 | 936 | 54.1 |
| Adults | 894 | 20.7 | 700 | 7.7 | 163 | 1.9 | 3312 | 69.8 |
| Senior adults | 105 | 20.0 | 52 | 4.3 | 15 | 1.3 | 424 | 74.4 |
| **Total** | 1463 | 18.08 | 2599 | 14.14 | 445 | 2.60 | 6001 | 65.18 |

*Children defined as less than age 10 years, adolescents and young adults defined as age 10-17 years, adults defined as age 18-64 years old, and senior adults defined as age 65 years and over.

^β^Infection with SARS-CoV-2 was defined by the presence of antibodies only based on the Luminex xMAP SARS-CoV-2 Multi-Antigen IgG assay algorithm.

^α^All percentages are weighted.
