# Supplementary material for: Seroprevalence of SARS-CoV-2 in four states of Nigeria in October 2020: A population-based household survey
Source: PLOS Glob Public Health. 2022 Jun 17;2(6):e0000363. doi: 10.1371/journal.pgph.0000363 (PMC10022353; doi:10.1371/journal.pgph.0000363)
Supplement: S1 Acknowledgments — (DOCX) [file pgph.0000363.s003.docx]

**S1 Acknowledgements**

The Nigeria SARS-CoV-2 Survey Group includes Principal Investigators: Elsie Ilori (NCDC), Rosemary Audu (NIMR), Laura Steinhardt (CDC Atlanta), Kristen A. Stafford (University of Maryland, Baltimore); Co-Investigators: Chikwe Ihekweazu, Nwando Mba, Ehimario Igumbor, Chinwe Ochu, William Nwachukwu, Chioma Dan-Nwafor, Sandra Mba, Ahmed Ladan, Rabiatu Aliyu Ahmed, Emeka Ndodo, Jafiya Abubakar, Ibrahim Zainab, Adesuyi Omoare, Ahumibe Anthony, Nasir Ahmed, Robinson Nnaji, Chimaobi Chukwu, Zaidat Musa, Fehintola Ige, Yohhei Hamada, Abideen Salako, Bisola Adebayo, Kikelomo Wright, Oliver Ezechi, Molebogeng Rangaka, Ibrahim Abubakar, Babatunde Salako, Kolapo Usman, Bala Mohammed Audu, Emem Iwara, Sylvia Adebajo, Manhattan Charurat, Akipu Ehoche, Natalia Blanco, Andrew Mitchell, Mirna Moloney, Alash’le Abimiku, Augustine Mpamugo, Henriatta Tiri, Dawit Ayele, Matthias Alagi, Orji Bassey, Curtis Blanton, Dalhatu Ibrahim, Stacie M. Greby, Oladipupo Ipadeola, Nnaemeka C. Iriemenam, Stephen McCracken, McPaul Okoye, Olumide Okunoye, Mashesh Swaminathan; Field Implementation, Laboratory and Data Management: Chinedu Agbakwuru, Baffa Sule Ibrahim, Stephen Ohakanu, Chukwuka Ezekwe, Akipu Ehoche, Samuel Indyer, Samuel Nwafor, Adepoju Adekemi, Gladys Antonza, Abisinuola Lawal, Rukevwe Aliogo, Edewede Onokevbagbe, Temitope Olaleye, Ifanyichukwu Odoh, Mudiaga Esiekpe, Ayuba Dawurung, Obinna Nnadozie, Ubong Okon, Yahaya Lamino, Sagir Awuaal, Promise Eneze, Isaac Egbelefu, Richard Adegbola, Azuka Okwuraiwe, Gregory Ohinhoin, David Oladele, Ifeoma Idigbe, Tajudeen Bamidele, Hussein Abdurrazaq, Basit Baruwa, Oluwagbemiga Aina, Toyosi Raheem, Adewale Ojogbede, Osuolale Qousim, Olufemi Amoo, Mabel Uwandu, Maureen Aniedobe, Chinyere Ezeudu, Liman M. Usman, Bile Nuhu
